# Is end-stage lateral osteoarthritic knee always valgus? Mechanical alignment analysis and radiographic severity assessment

**DOI:** 10.1007/s10195-015-0356-9

**Published:** 2015-06-03

**Authors:** Su Chan Lee, Viral Gondalia, Byoung Yoon Hwang, Hye Sun Ahn, Choon Key Lee, David J. Hunter, Kwang Am Jung

**Affiliations:** Joint and Arthritis Research, Department of Orthopaedic Surgery, Himchan Hospital, 20-8, Songpa-dong, Songpa-gu, Seoul, 138-170 Korea; Rheumatology Department, Royal North Shore Hospital and Northern Clinical School, University of Sydney, Sydney, NSW Australia

**Keywords:** Lateral compartment, Osteoarthritis, Knee

## Abstract

**Background:**

We hypothesized that not all persons with end-stage lateral osteoarthritis (OA) have valgus malalignment and that full extension radiographs may underreport radiographic disease severity. The purpose of this study was to examine the demographic and radiographic features of end-stage lateral compartment knee OA.

**Materials and methods:**

We retrospectively studied 133 knees in 113 patients who had undergone total knee arthroplasty between June 2008 and August 2010. All patients had predominantly lateral idiopathic compartment OA according to the compartment-specific Kellgren–Lawrence grade (KLG). The mechanical axis angle (MAA), compartment-specific KLG and joint space narrowing (JSN) of the tibiofemoral joint at extension and 30° of knee flexion, tibia vara angle, tibial slope angle, body mass index, age, and sex were surveyed.

**Results:**

End-stage lateral compartment knee OA has varus (37.6 %), neutral (22.6 %), and valgus (39.8 %) MAA on both-leg standing hip-knee-ankle radiographs. KLGs at 30° of knee flexion (fKLG) were grades 3 and 4 in all patients. However, for KLGs at full extension (eKLG), 54 % of all patients had grades 3 and 4. The others (46 %) showed grades 1 and 2. We observed significant differences in lateral compartment eKLG/eJSN (2.3/2.3 mm in varus, 2.5/1.9 mm in neutral, 2.9/1.6 mm in valgus, *p* = 0.01 and 0.03, respectively), tibia vara angle (4.9° in varus, 4.1° in neutral, 3.0° in valgus, *p* < 0.01), and medial compartment eKLG/eJSN (2.1/3.1 mm in varus, 2.0/3.4 mm in neutral, 1.8/4.3 mm in valgus, *p* < 0.01 and 0.01, respectively) between MAA groups, except for the tibial slope angle (9.7° in varus, 10.1° in neutral, 9.8° in valgus, *p* = 0.31).

**Conclusion:**

Varus alignment was paradoxically shown in approximately one-third of those with end-stage lateral knee OA on both-leg standing hip-knee-ankle radiographs. Films taken in full extension underreported the degree of OA radiographic severity.

**Level of evidence:**

Level IV, observational study.

## Introduction

Osteoarthritis (OA) in the knee joint is the most common disorder in orthopedics and is characterized by structural and functional failure of the synovial joint tissue with loss and erosion of articular cartilage, subchondral bone alteration, meniscal degeneration, and bone and cartilage erosion [1].

Conventional radiography is the most convenient and important imaging examination in a clinical setting when evaluating a patient who has a known or suspected diagnosis of OA. Radiographs clearly visualize bony features, including marginal osteophytes, subchondral sclerosis, and bone cysts, but provide only an estimate of cartilage thickness and meniscal integrity by joint space narrowing (JSN). However, progression of JSN is the most commonly used criterion for the assessment of OA progression, and the complete loss of joint space width, characterized by interbone contact is one of the factors considered in the decision for joint replacement.

Radiography is an indispensable complement to clinical examination and plays a key role in diagnosing and monitoring the course of a condition in OA. Lower limb alignment and extended and/or semi-flexed knee anteroposterior radiographs can be used for evaluating the relationship between OA and compartmental pattern and severity of knee OA.

However, most studies have focused on patients with early stage OA. In addition, there is insufficient knowledge for lateral compartmental OA in comparison to medial compartment OA. This may raise the question of whether similar findings from different radiographic methods are found in end-stage lateral compartment OA. Better understanding of the radiologic characteristics of end-stage lateral OA will be helpful in diagnosing and managing some patients with advanced disease. The purpose of this retrospective study was to examine the demographic and radiographic features of end-stage lateral compartment knee OA (Kellgren–Lawrence grade 3 or 4). We hypothesized that not all persons with end-stage lateral OA have valgus malalignment and that full extension radiographs may underreport radiographic disease severity.

## Materials and methods

We retrospectively reviewed the records of 133 patients who had undergone primary total knee arthroplasty between June 2008 and August 2010. All subjects had lateral compartment OA based on the following criteria: (1) only lateral compartment involvement, (2) only lateral and patellofemoral involvement, or (3) involvement of the lateral and medial compartments (with or without patellofemoral involvement) but with the lateral involvement more severe than the medial involvement according to the compartment-specific Kellgren–Lawrence grade (KLG) [2, 3].

**Fig. 1:**
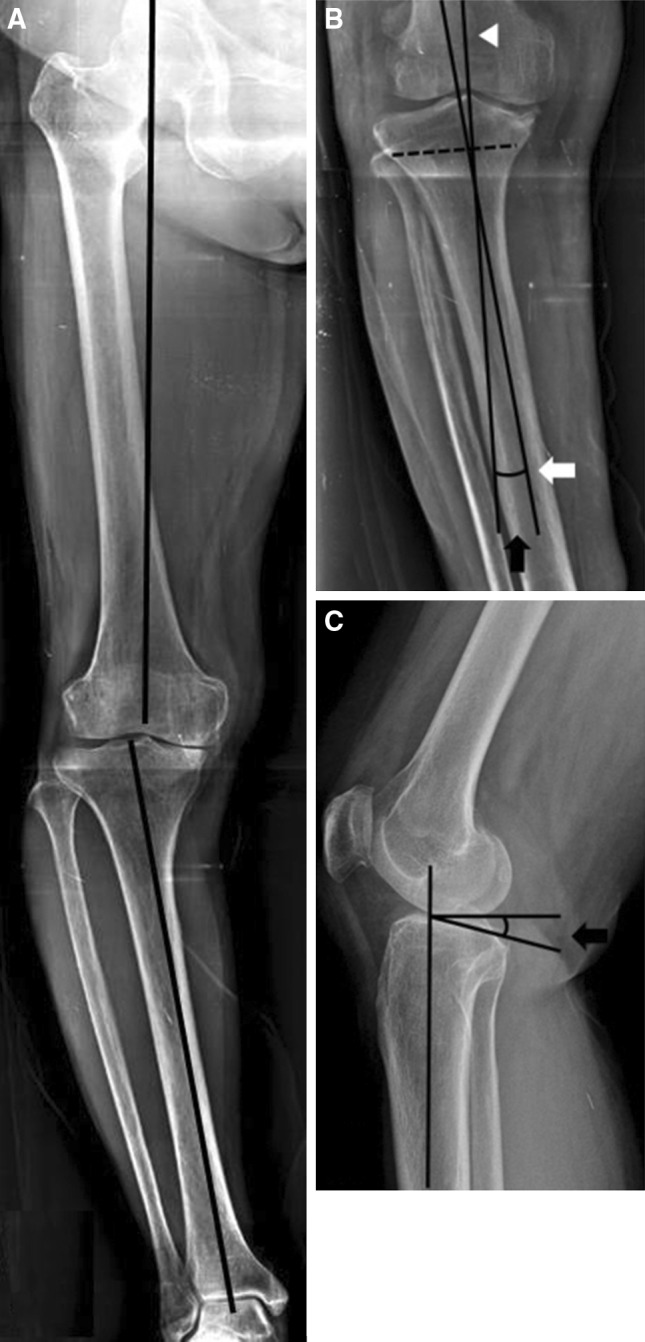
Mechanical axis angle is the angle between a line from the center of the femoral head to the mid-condylar point between the cruciate ligaments and a line from the center of the tibial plateau to the center of the tibial plafond (**a**). The tibia vara angle (*black arrow*) is formed by the line perpendicular to the epiphysis (*white arrowhead*) and the anatomical axis of the tibia (*white arrow*) (**b**). The posterior tibial slope angle is defined as 90° minus the angle made by the intersection of the line along the longitudinal axis of the tibia and the slope of the medial tibial plateau (**c**)

A detailed retrospective review of the medical records of these patients was conducted to extract all pertinent information on the body mass index and gender. Preoperatively, standing hip-knee-ankle radiographs were taken and the mechanical axis angle (MAA) was measured. MAA is the angle between a line from the center of the femoral head running distally to the mid-condylar point between the cruciate ligaments (femoral mechanical axis) and a line from the center of the tibial plateau extending distally to the center of the tibial plafond (tibial mechanical axis) [4]. The neutral MAA was categorized as 0° to 2° of varus. The tibia vara angle was defined as the angle between a line perpendicular to the epiphysis and the anatomical axis of the tibia, which was measured using radiographs of the entire lower limb. The line to the epiphysis was measured perpendicular to the line that connected both ends of the epimetaphy seal junction (Fig. 1) [5–10]. Compartment-specific KLG and JSN were measured at extension and 30° of knee flexion on weight-bearing views. Compartment-specific JSN was measured from the center of the medial/lateral condyle to the center of the medial/lateral tibial plateau [11]. To assess reliability, each evaluation (KLG, MAA, tibia vara angle, and tibial slope angle) was measured by two experienced researchers (BYH, HSA) under the supervision of the coauthor (KAJ, with 10 years’ musculoskeletal radiology experience), who were blinded to patients’ information using the PACS system (INFINITT Healthcare Co Ltd, Seoul, Korea). The average of the two individual mean values was used.

### Statistical analysis

SPSS (Statistical Package for the Social Sciences, v.12.0, Chicago, IL) was used for statistical analyses. For tibia vara angle and tibial slope angle (continuous data), and JSN, ANOVA was used to analyze differences in variables with differing MAA. For comparing KLG (categorical ordinal data), a chi-square test was employed. Bivariate analysis (Spearman’s correlation coefficient for categorical data) was used to determine the correlation between radiologic mismatches and variables. The interobserver reliability in measuring variables (KLG, MAA, tibia vara angle, JSN, and tibial slope angle) was evaluated using the intraclass correlation set at a 95 % confidence interval. A level of significance was set at *p* < 0.05.

## Results

There were 119 out of 133 knees from females. End-stage lateral compartment knee OA has varus (37.6 %), neutral (22.6 %), and valgus (39.8 %) MAA on both-leg standing hip-knee-ankle radiographs. KLG at 30° of knee flexion (fKLG) was grades 3 and 4 in all patients. However, for the KLG at full extension (eKLG), 54 % of all patients had grades 3 and 4. The others (46 %) showed grades 1 and 2, which caused mismatches of KLG between extension and 30° of knee flexion (radiologic mismatch). Only the MAA had a negative correlation with radiologic mismatch (*r* = −0.486, *p* < 0.01) (Table 1). With a more valgus MAA, there was less radiologic mismatch.

**Table 1:** Correlation coefficient between radiologic mismatch and other factors

	Pearson’s correlation coefficient	*p* value
Age, years	0.096	0.29
Sex, M/F	0.014	0.88
Body mass index, kg/m^2^	−0.058	0.58
Mechanical axis angle, °	−0.486	<0.01
Tibia vara angle, °	0.196	0.12
Tibial slope angle, °	0.109	0.23
Lateral compartment joint space width at extension, mm	0.286	<0.01
Medial compartment joint space width at extension, mm	−0.129	0.14
Lateral compartment joint space width at flexion, mm	0.002	0.98
Medial compartment joint space width at extension, mm	0.152	0.08

We observed significant differences in lateral compartment eKLG/eJSN (2.3/2.3 mm in varus, 2.5/1.9 mm in neutral, 2.9/1.6 mm in valgus, *p* = 0.01 and 0.03, respectively), tibia vara angle (4.9° in varus, 4.1° in neutral, 3.0° in valgus, *p* < 0.01), and medial compartment eKLG/eJSN (2.1/3.1 mm in varus, 2.0/3.4 mm in neutral, 1.8/4.3 mm in valgus, *p* < 0.01 and 0.01, respectively) between MAA groups except for the tibial slope angle (9.7° in varus, 10.1° in neutral, 9.8° in valgus, *p* = 0.31) (Table 2). The post-hoc test showed increased mean lateral compartment eKLG/eJSN in valgus MAA compared to the others, increased tibia vara angle in varus MAA compared to valgus, increased medial compartment eKLG/eJSN in varus, and neutral MAA compared to valgus.

**Table 2:** Demographic and radiologic features of end-stage lateral knee osteoarthritis with differing alignment

	Varus (*n* = 50)	Neutral (*n* = 30)	Valgus (*n* = 53)	*p* value
Mean age (range), years	69.3 (57–80)	69.3 (60–79)	68.1 (57–83)	0.60
Sex, M/F	4/46	3/27	7/46	0.62
Body mass index, kg/m^2^	25.1 ± 3.3	26.2 ± 2.8	25.8 ± 3.2	0.44
Lateral compartment Kellgren–Lawrence grade at extension	2.3 ± 0.7	2.5 ± 0.8	2.9 ± 0.8	<0.01
Lateral compartment joint space width at extension, mm	2.3 ± 0.8	1.9 ± 0.9	1.6 ± 0.8	0.03
Radiologic mismatch, %	50.0	60.0	84.9	<0.01
Medial compartment Kellgren–Lawrence grade at extension	2.1 ± 0.4	2.0 ± 0.4	1.8 ± 0.5	0.03
Medial compartment joint space width at extension, mm	3.1 ± 1.2	3.4 ± 1.0	4.3 ± 0.9	0.01
Tibia vara angle, °	4.6 ± 2.7	3.8 ± 2.3	2.7 ± 2.9	<0.01
Tibial slope angle, °	9.7 ± 3.1	10.1 ± 2.8	9.8 ± 3.2	0.26

Among all patients, 20 patients (15.0 %) had bilateral severe lateral OA, and 47 patients (35.3 %) had contralateral severe medial OA who underwent TKA. There was no difference in BMI or sex between the two groups. However, in the former group, significant older age and increased mismatch was observed.

The intraclass correlation coefficient for inter-tester reliability of KLG, MAA, tibia vara angle, joint space width (JSW), and tibial slope angle was 0.785, 0.833, 0.802, 0.753, and 0.812, respectively.

## Discussion

This study focused on the radiologic and demographic features of end-stage lateral knee OA. In this study, all patients showed grades 3 and 4 KLGs in their flexion view, representing bone to bone contact (end-stage). However, not all patients showed grades 3 and 4 KLGs on extension views. Valgus and neutral MAA accounted for the majority of our sample with end-stage lateral knee OA. Varus alignment was also paradoxically shown in approximately one-third of those with end-stage lateral knee OA.

Radiographic protocols of the knee in flexion have been shown to improve the detection of JSN by providing better exposure of the location of the greater cartilage changes in the posterior area of the femoral condyles [11–14]. The flexion weight-bearing radiograph is commonly used and is reportedly markedly better than the conventional radiograph in evaluating detection of JSN and disease severity [15, 16]. The contact zones of femorotibial articulation shift in both area and location as flexion occurs. As the knee is flexed during the stance phase of gait, the femorotibial contact area moves posteriorly and decreases in size. With greater loads per unit of area, the cartilage is more susceptible to degeneration in the contact zones of flexion. Because of this, the sensitivity and specificity of the flexion weight-bearing radiograph is markedly better than the conventional extension radiograph [15, 16]. In other words, the extension view risks underestimation in diagnosing OA compared to the flexion view.

Despite the generalized loss of articular cartilage in end-stage osteoarthritis, JSN is not always found to be consistent between extension and flexion weight-bearing views, contributing to radiologic mismatch. Our study showed that 46 % of patients with end-stage lateral OA showed this phenomenon.

Meniscal damage also has an important role in OA. The vast majority of meniscal tears occur in their posterior half and thus chondral damage and loss of joint space occurs when the knee is loaded in the flexed position. Anteriorly, the meniscus and articular cartilage is usually intact, and so in the extended position there is less loss of joint space. Due to a stance phase knee adduction moment, even during normal gait in healthy knees, more load passes through the medial tibiofemoral compartment than through the lateral compartment [17, 18]. For this reason the extension view is more suited for observing medial compartment OA than lateral disease. However, limb alignment becomes more valgus angulated with an increase in flexion rather than extension [19], which distributes more weight in the lateral compartment and shows lateral JSN in knee flexion.

Previous reports noted that lower extremity malalignment increases the rate of progression of knee osteoarthritis [20–22]. An increase in the varus angle was associated with a significantly increased adjusted risk of having severe medial disease. Also, valgus alignment increases the risk of progression of lateral disease, and an increasing valgus angle is associated with more severe progression of lateral disease [2, 20, 21]. However, in our study, a large proportion of patients with end-stage lateral OA showed varus and neutral alignment. Indeed ‘varus’ alignment was shown in approximately one-third of patients with end-stage lateral knee OA on both-leg standing hip-knee-ankle radiographs. However, it is clear that lateral cartilage loss is advanced by ‘valgus’ alignment during walking. The knee which is originally valgus is simply seen to be ‘varus’ on both-leg standing hip-knee-ankle radiographs, but it is not ‘varus’. Brouwer et al. [22] reported the prevalence of malalignment in knees without OA in 2290 knees, and observed 25 % with varus alignment, and 36 % with valgus alignment. Interestingly, our study showed that in end-stage lateral OA, the distribution of valgus malalignment was similar to the normal knees proportion.

Both knees demonstrated end-stage lateral OA (knock knees) in 15 % of all patients. Of enrolled patients who showed contralateral medial OA, 35.3 % underwent TKA (windblown knees). The patients with contralateral medial compartment OA all had varus MAA in the contralateral lower limb. There was no difference in BMI or sex between knock and windblown knees. The former group showed older age than the latter. This suggested that varus alignment would affect the progression of medial compartment OA more, compared to the valgus alignment effect on lateral compartment OA. Brouwer et al. [22] observed a borderline effect of valgus malalignment on the risk of incident OA, while varus malalignment had a larger effect.

Some reported that limb alignment modulates the effect of standard risk factors for progression of OA of the knee, including obesity, quadriceps strength, laxity, and stage of disease [23–25]. In medial compartment OA, limb alignment has a great effect on OA prevalence. However, in lateral OA, another factor beside limb malalignment would have more effect on the lateral compartment than the medial compartment.

This study did have some limitations. First, we performed only a cross-sectional observational study. Second, we attempted to control for this by grouping grades 3 and 4 together (severe disease) and grades 1 and 2 together (mild to moderate disease). In this way, differences between severe and mild to moderate disease are much more likely to indicate a real change in radiographic evidence of disease progression than would have been the case if each of the four grades had been considered separately. Third, enrolled patients in this study were all symptomatic. The symptoms of OA of the knee are typically described as mechanical—that is, they occur with physical activity. Fourth, this study was based on X-ray findings, not on MRI. JSW is determined not only by cartilage thickness, but also other factors such as knee angle, direction of the X-ray beam, and meniscal status. We thought that further study based on MRI would enable assessment of the articular cartilage without being affected by any of these, and could be helpful in uncovering the reason why such mismatch occurs.

Valgus and neutral MAA accounted for the majority of our sample with end-stage lateral knee OA on the both-leg standing hip-knee-ankle radiographs. Varus alignment was also paradoxically shown in approximately one-third of those with end-stage lateral knee OA. Radiographs taken in full extension underreported the degree of OA radiographic severity, with more mismatch being evident with more varus alignment. Varus MAA showed positive correlations with increased tibial vara angle and medial compartment eKLG in end-stage lateral OA.
